# Therapeutic interventions in Australian primary care, youth mental health settings for young people with borderline personality disorder or borderline traits

**DOI:** 10.1186/s40479-020-00138-2

**Published:** 2020-10-01

**Authors:** Nikki O’Dwyer, Debra Rickwood, Dean Buckmaster, Clare Watsford

**Affiliations:** Faculty of Health, University of Canberra, Canberra, ACT 2601 Australia

**Keywords:** Youth mental health, Borderline personality disorder, Cognitive behavior therapy, Early intervention

## Abstract

**Background:**

This study aimed to investigate what therapeutic interventions were being applied by clinicians working with young people with a diagnosis of Borderline Personality Disorder or borderline traits in Australian primary mental health care settings. Given the current lack of evidence-based guidelines for treatment with this client population, investigating what is being implemented is needed. The study also aimed to determine whether the interventions clinicians are using are effective in reducing distress and increasing functioning for these clients.

**Methods:**

Participant data came from the national minimum data set for *headspace* youth mental health centers across Australia. Young people’s data were included in the study if the young person was diagnosed with Borderline Personality Disorder or borderline traits during their first episode of care (*N* = 701). Clinician data that indicated the type of intervention used at each client session and outcome measures routinely captured were analyzed to determine interventions used and outcomes achieved.

**Results:**

Results demonstrated that CBT was the most frequently used modality of intervention followed by supportive counselling and IPT, but that most clients received a variety of intervention types. There were no or only weak relationships between changes in outcomes and the amount of any type of intervention that was provided. No significant relationship was found with the amount of CBT a client received and changes in symptoms or functioning, despite being the most commonly employed modality.

**Conclusions:**

The study highlights the need for evidence-based treatment guidelines for early intervention in young people with borderline personality disorder traits.

## Background

Like most mental disorders, Borderline Personality Disorder (BPD) usually emerges during adolescence and early adulthood [32]. Given the significant emotional, functional and economic impact of this diagnosis [7, 22], the need for evidence-based early intervention for young people is critical. While there is an emerging evidence-base for adolescents and younger clients, no treatment guidelines are currently available and understanding what clinicians currently do to intervene in young people’s lives who meet the criteria for BPD is an important step to inform greater efficacy in treatment.

### Onset and prevalence of borderline personality disorder

Personality disorders are characterised by an on-going pattern of behaviour and inner experience that generally becomes evident in adolescence or early adulthood, remains stable over time, and causes decreasing functioning and increasing distress [1]. One of the most commonly diagnosed personality disorders is BPD. To make a diagnosis in adolescence a clinician is required to assess that the maladaptive personality traits have been pervasive and persistent for at least a year, and are considered unlikely to be limited to a normal developmental stage [1].

Historically, clinicians and services have been reluctant to diagnose and treat younger clients [6]. This reluctance was associated with a presumption that personality is still forming in adolescence and that there is stigma associated with the diagnosis necessitating a cautious approach [15]. Consequently, terms like “emerging BPD”, which avoid attributing a diagnosis, have become common. However, research shows that diagnosis of BPD in adolescence is valid, reliable, and predictive of serious distress and dysfunction over subsequent decades [32]. It has been argued that since evidence shows that BPD symptoms that emerge in adolescence persist into adulthood, the diagnosis should be given and that a failure to do so likely results in ineffective treatment [9].

Despite prior clinician reluctance to diagnose BPD in those under 18 years, it is estimated that 1–3% of young people under 18 years meet the criteria [34]. Within clinical populations, 33–49% of adolescent inpatients are believed to meet criteria for BPD, and 11–22% of adolescents being treated as outpatients. The prevalence rates in adolescence are similar to or higher than those reported for adults [18]. Zanarini et al. [42] demonstrated that while patients with BPD most frequently start psychotherapy after the age of 18 years, their symptoms usually started much earlier. Of particular concern, and highlighting the need for early intervention, this research, undertaken in the United States, found that 30% were self-harming prior to 12 years of age, with another 30% initiating self-harm between 13 and 17 years of age.

### Impact of borderline personality disorder

Along with the likely onset of BPD during adolescence, the need for evidence-based early intervention is further supported by the established impact it has on both the individual and the community as a whole. In a study 351 young adults aged 18 to 24 years, features of BPD predicted poorer academic achievement, social maladjustment and poorer overall functioning when compared with peers at two-year follow-up [4]. The pervasive nature of BPD symptoms are the reason clients meeting this diagnostic classification struggle in fulfilling roles like being a partner, a student or an employee.

Of major concern is the research indicating a diagnosis of BPD impacts on life expectancy. Research in Scandinavia of 270,770 patients with recent onset mental disorders found that those with BPD had a life expectancy 15 to 20 years shorter than the general population [33]. This was related to suicide and increased medical issues (cardiovascular disease, metabolic conditions, and respiratory disease). For those with BPD, suicide and self-harm are particular risk factors affecting morbidity and mortality. For example, Goodman et al. [20] found in a sample of 104 adolescents and 290 adults with a diagnosis of BPD that 90% engaged in self-mutilation and 75% had made multiple suicide attempts.

### Evidence-based interventions for borderline personality disorder in young people

Health professionals have an ethical obligation to provide services that have an evidence base, and the Australian Psychological Society (APS) recommends Dialectical Behavioural Therapy (DBT) and Mentalisation-based Therapy (MBT) as the “standout” interventions for adults with BPD [3]. It also concludes that Cognitive Analytic Therapy (CAT), Cognitive Behavioural Therapy (CBT) and Schema-focused Therapy (SFT) can be therapeutically indicated for less severe BPD symptomology.

The APS review notes a current gap in the evidence for BPD treatments for adolescents and young people compared with the evidence for adults. It found only Level III-3 evidence (a comparative study without concurrent controls) for CAT and Level IV evidence (case series with pre test/post test outcomes) for DBT. A psycho-dynamic approach was also found to have a Level IV evidence base for adolescents [3].

The first systematic review and meta-analysis of RCTs for psychotherapies for adolescents with BPD has been reported recently [41]. It found only seven studies to include in the meta-analysis and concluded that overall, psychotherapies had significant and large effects on BPD-specific symptomatology and the frequency of non-suicidal self-injury, in the short term, but that the efficacy of treatments was not statistically significant at long-term follow-up. It was noted that these findings are similar to those for the adult literature, but there were too few studies to compare different types of psychotherapy (e.g., DBT vs CBT).

MBT and DBT have been shown to have the greatest impact on suicidality and self-harm in adolescent clients seeking early intervention [18]. MBT assumes that the development of BPD in adolescence is grounded in a phase-specific compromise in the capacity to mentalise or think about thinking [5]. An adaptation for adolescents (MBT-A) has been developed which incorporates monthly family sessions to the treatment regime, given that most adolescents still live at home. A randomised control study was conducted on a sample of 12–17 year olds who had at least one episode of self-harm [38]. The 12 month intervention demonstrated a significant decrease in number of suicide attempts, self-harming behaviour and depression compared with the treatment as usual condition.

DBT has been adapted for adolescent clients (DBT-A) incorporating family sessions, reducing the length of treatment and simplifying the skills to be more developmentally appropriate [36]. A review of 18 studies examining the empirical outcomes of DBT interventions with adolescents has been conducted [28]. The evidence concluded an overall positive treatment effect to address suicidality (*d =* 0.73), depression (*d =* 0.76) and BPD symptomatology (*d =* 0.65). Additionally, Mehlum et al. [31], in a randomized trial with adolescents, compared enhanced usual care with DBT, finding DBT was superior in reducing self-harm, severity of suicidal ideation and depression. For older youth aged 18 to 25, when compared with treatment as usual, DBT showed greater reductions in suicidality, depression, number of NSSI events, BPD criteria and psychotropic medication use [35]. Although DBT-A is emerging as a recommended treatment for adolescents with BPD, it is not an early intervention model and requires a commitment to participate in intensive multi-modal treatment. This comprehensiveness could limit access for less severe clients indicated for early intervention.

CBT has a Level I evidence base for the greatest number of disorders in the DSM-5 [3]. For BPD in young people, however, the evidence is not strong, comprising only Level III evidence, the second lowest rating. A randomised control study of adult clients with BPD receiving traditional CBT (up to 30 sessions) compared with treatment as usual, appeared to be beneficial for symptoms of depression, anxiety and negative cognition [14]. The study found, however, that a traditional CBT approach did not decrease the number of emergency contacts, hospitalisation, self-harm or improve functioning generally. A quasi-experimental design found that an adaption of CBT, named Cognitive Analytic Therapy (CAT), resulted in the reduction in externalising psychopathology, such as intense anger outbursts and disinhibited behaviour, compared with baseline [8]. Given the fundamental aspects of identity formation and relationship experiences for people with BPD, it has been suggested that traditional CBT approaches, focusing on specific cognitions and behaviours, fail to address the fundamental aspects of the disorder, which are emotional regulation, interpersonal relationships volatility, and suicidal and self-injurious behaviours [27].

It is clear that for younger populations the evidence is currently not strong regarding the best treatments for BPD, although some modalities appear promising, including DBT-A and CAT. Given the major impact of this diagnosis on young people, better evidence to inform treatment decisions is much needed to reduce the negative effects of psychopathology, improve function and reduce risk. Investigating current practice in early intervention services for young people at risk of BPD may inform future directions in research and guideline development.

### Aims

In the absence of clear treatment guidelines for practice, the current study aimed to investigate the therapeutic interventions being used by clinicians working in early intervention services treating young people with a diagnosis of BPD or identified traits of BPD in an Australian context. The study aimed to determine whether the most common interventions used by clinicians were effective in reducing psychological symptoms and improving functioning for these clients. It was hypothesised that the therapeutic interventions used would not be well-informed by evidence, meaning that a wide range of interventions would be employed, and that CBT would be the most common treatment approach (as it has the strongest evidence base generally and most clinicians are trained in this approach). Secondly, it was hypothesised that CBT would not result in a significant improvement in psychological distress or functioning for clients attending an early intervention service with a diagnosis of BPD.

## Methodology

### Participants

Participants were young people who had attended a *headspace* youth mental health centre. *Headspace* is the Australian Government’s national youth mental health initiative, which since 2006 has rolled out easily accessible mental health service centres designed specifically for young people aged 12 to 25 to assist with their mental health, health and wellbeing needs [29]. Participant data came from the *headspace* national minimum dataset (MDS) for the four-year period from 1 April 2013 to 31 March 2017. *headspace* collects a MDS for all clients accessing headspace centre services [37]. For this time period, the national dataset comprised information on 74,804 young people who were presenting for the first time at 76 *headspace* centres. For the current study, only young people presenting with a primary issue of ‘Borderline Personality Traits’ at intake or first assessment were selected (*N =* 701).

Participants were aged between 12 and 25 years. The mean age was 19.68 years (SD = 2.76). The majority were female (80.7%), 11.1% were male and 2.1% described themselves as ‘other’; data were missing for 6.0%. Participants came from all Australian states and territories with 44.7% from Victoria, 15.3% from New South Wales, 18.5% from Queensland, 13.7% South Australia, 5.7% from Western Australia, 1.0% from Tasmania, 1.0% from the Australian Capital Territory and, 0.1% from the Northern Territory.

### Procedure

The *headspace* MDS is collected from each client and service provider at every occasion of service. Upon initial presentation to a centre and at each subsequent occasion of service, clients are given either an iPad or access to a private computer to complete an electronic survey which takes approximately 15 min to complete. Service providers also provide relevant information from each occasion of service in an electronic form. Data are encrypted to ensure confidentiality and stored in a national data warehouse. Clients consent for their de-identified information to be used for service evaluation and research purposes. Approval for the MDS was obtained from the Clinical and Research Board sub-committee and data usage is overseen by the Data Governance Group.

For the current study, data were extracted for clients who were presenting for their first occasion of service and only clients who received a diagnosis of a personality disorder with ‘Borderline Traits’ during their treatment at *headspace* were included. For service provider data, only data from clinicians who provided mental health care were included, comprising psychologists (who were the predominant group), psychiatrists, psychiatric registrars, social workers and mental health nurses. Data from medical visits, youth work sessions, alcohol and other drug services, and vocational services were excluded.

### Measures

The following measures were extracted from the minimum dataset.

#### Client characteristics

##### Demographics

Participants’ age in years, gender, Aboriginal and/or Torres Strait Islander identity, sexual orientation and level of education were extracted. These data are self-reported by young people attending headspace.

##### Diagnosis of BPD or presentation with BPD traits

At each occasion of service, the service provider is asked to provide information about the young person and the services provided. This includes determining the main presenting issues for the young person and whether they have a diagnosable mental disorder. Primary diagnosis is determined from a list of 21 subcategories following the broad classifications of the DSM-5 [1]. Clinicians provided a diagnosis according to their usual practice as assessed during sessions with clients. Clinicians are also able to rate the diagnosis of the young person as Not applicable or Diagnosis not yet assessed.

#### Service provision

##### Level of service engagement

The number of *headspace* appointments participants attended was used to measure level of engagement with mental health services.

##### Type of intervention

At each occasion of service clinicians are asked to assign a service type for the session they provided. Clinical interventions included: assessment, Cognitive Behavioural Therapy, behavioural intervention, supportive counselling, crisis support, psycho-education, Interpersonal Therapy, Acceptance and Commitment Therapy, Mindfulness based therapy, motivational interviewing, Narrative Therapy, and Other.

#### Outcome measures

##### Psychological distress

Psychological distress was measured using the Kessler – 10 (K10), which measures depressed mood, hopelessness, restlessness, fatigue, nervousness and worthlessness for the last 4 week period [25]. Item statements (e.g. “About how often did you feel tired out for no good reason?”) were rated on a five-point response scale from ‘none of the time’ to ‘all of the time’. Items are summed to provide a total score ranging from 10 to 50, with higher scores reflecting greater distress. A score on the K-10 of 22–29 indicates high levels and of 30–50 indicates very high levels of distress [2]. The K-10 has shown excellent internal consistency and reliability with Cronbach’s alpha = .93 [25].

##### Social and occupational functioning

Service providers rate level of functioning using the one-item Social and Occupational Functioning Assessment Scale (SOFAS). It is derived from the DSM-IV Global Assessment of Functioning Scale, which has shown good internal consistency and reliability with Cronbach’s alpha = .80 [19]. The service provider rates a client from 1 to 100, from being unable to function and maintain minimal personal hygiene (a rating of 10 or less), moderate difficulty in social, occupational or school functioning (51–60), to superior functioning in a wide range of activities (91–100).

##### Quality of life

Quality of life was measured by the Brief Multidimensional Students’ Life Satisfaction Scale (LSS) [40], which measures self-reported quality of life in seven different domains: for family life, friendships, romantic relationships, school/work experience, yourself, where you live, and life overall. Answers are given on a scale from 0 to 10 with 0 being the worst level of satisfaction and 10 being the best possible. A total score was derived by averaging across the seven domains. A score of 6 or more indicates a positive level of life satisfaction [13].

### Statistical analysis

First, the percentage of clients receiving each type of intervention at each of the first 10 service sessions was calculated. Ten sessions was selected as the cut-off point because this is the number of mental health sessions funded per year under the Australian Medical Benefits Schedule; although some clients received many more than 10 sessions, the numbers drop off markedly and reliability of estimates is greatly reduced. The number of clients who received each type of intervention overall was also calculated.

For analysis of outcomes, change scores were calculated for the K10, SOFAS, and LSS by subtracting the first score for the client’s initial visit from the final measurement taken at their last recorded session within the time period. Changes were categorised as improved, no change, or got worse. The total change scores were correlated with the amount of each type of intervention reported.

## Results

Data were analysed using SPSS v23 [24].

### Interventions

Participants received between 1 and 30 sessions. Note that headspace data only collects information up until the 30 session mark. The average number of sessions was 6.22 (SD = 6.86), but was highly skewed (skew = 1.889, SE = .092). There were 81% who received between 1 and 10 sessions. The average number of sessions for those who received up to and including 10 sessions, excluding those who received more than 10 sessions, was 3.44 (SD =2.64), and the skew was not pronounced (skew = .896, SE = .103).

**Fig. 1:**
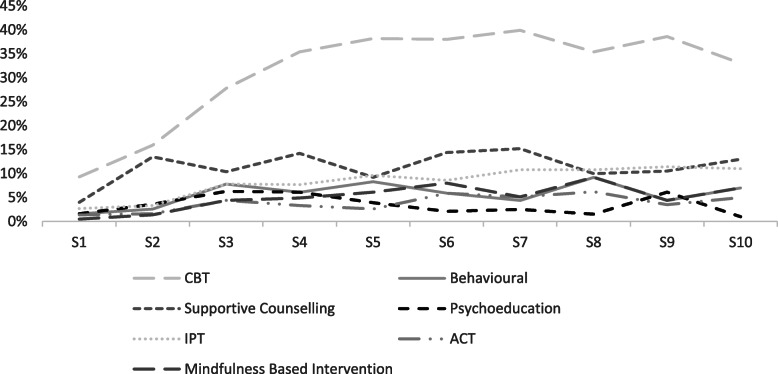
Percentage of clients receiving each intervention type over time (sessions 1–10)

Table 1 presents the percent of clients receiving each type of intervention at each of the first 10 sessions. This showed that three quarters of participants received an assessment at their initial session. There was another 22.6% that received a therapeutic session at visit 1, primarily CBT (9.3%). The proportion receiving assessment dropped off sharply over time and the percentage of clients receiving therapy increased. The most common therapeutic intervention by far was CBT with 39.9% receiving this intervention by session 7. After CBT, supportive counselling and IPT were the used most frequently used. These patterns are apparent in Fig. 1.

**Table 1:** Percentage of Clients Receiving Each Type of Intervention at Each Session Over First 10 Sessions

Intervention type	Session number									
	1	2	3	4	5	6	7	8	9	10
Assessment	74.9	30.8	21.5	13.8	9.2	3.7	3.8	2.3	5.3	5.0
CBT	9.3	15.9	27.8	35.4	38.2	38.0	39.9	35.4	38.6	33.0
Behavioral	1.6	2.6	7.8	6.1	8.3	5.9	4.4	9.2	4.4	7.0
Supportive Counselling	4.0	13.5	10.4	14.2	9.2	14.4	15.2	10.0	10.5	13.0
Crisis	0.3	1.4	0.7	0.8	1.3	2.1	2.5	0.8	1.8	0.0
Psychoeducation	1.6	3.6	6.3	6.10	3.9	2.1	2.5	1.5	6.1	1.0
IPT	2.7	3.3	7.8	7.7	9.6	8.6	10.8	10.8	11.4	11.0
ACT	1.4	1.6	4.4	3.30	2.6	5.9	5.1	6.2	3.5	5.0
Mindfulness	0.5	1.4	4.4	4.9	6.1	8.0	5.1	9.2	4.4	7.0
Motivational Interviewing	0.8	1.4	0.7	0.8	0.0	0.5	1.3	0.8	1.8	2.0
Narrative Therapy	0.3	0.2	0.4	0.8	0.4	0.5	0.0	0.0	1.8	0.0
Other	2.5	24.3	7.8	6.1	11	10.2	9.5	13.8	10.5	16.0

Table 2 shows the number of sessions, up to 10 sessions, that were received for each type of intervention. The most common again was CBT, however, only two clients received 10 sessions of CBT and nine clients received nine sessions. The next most common was IPT, with one client having nine sessions of this intervention. Third most common was supportive counselling, with three clients receiving eight sessions of this type of intervention.

**Table 2:** Number of Clients Receiving Each Number of Sessions by Type of Intervention

Intervention type	Number of sessions received									
	1	2	3	4	5	6	7	8	9	10
CBT	80	53	26	23	16	12	7	6	9	2
Behavioral	62	19	7	1	4	1	0	0	0	0
Supportive Counselling	73	33	13	9	6	5	5	3	0	0
Crisis	27	4	2	0	0	0	0	0	0	0
Psychoeducation	55	11	4	1	1	0	0	0	0	0
IPT	37	6	7	6	3	2	5	3	1	0
ACT	24	8	3	2	0	2	1	0	0	0
Mindfulness	36	11	3	5	1	1	0	1	0	0
Motivational Interviewing	24	5	0	0	0	0	0	0	0	0
Narrative Therapy	8	0	1	0	0	0	0	0	0	0
Other	131	52	31	20	8	6	6	2	1	1

### Outcomes

Table 3 shows the baseline mean outcome scores and percentage of clients who improved, had no change, or got worse for each of the change measures. The baseline scores show that these young people were very highly distressed at presentation; had moderate difficulty in social, occupational or school functioning; and demonstrated low levels of life satisfaction. The K10 change scores revealed that 60% of clients either got worse or had no change. On the SOFAS, more than half improved somewhat, but 45% had no change in scores or got worse. On the LSS measure, 68.9% of clients were worse or had no change in their quality of life.

**Table 3:** Percentage of Clients Showing Each Type of Change by Outcome Measure

Outcome	Baseline mean score (SD)	Type of change		
		Worse	No Change	Improved
K10	36.13 (7.89)	19.6	40.4	39.9
SOFAS	57.42 (11.77)	32.3	12.7	55.0
LSS	3.98 (1.74)	22.8	46.1	31.1

Table 4 shows the association between the amount of each type of intervention and the outcomes on the change scores. This reveals that the only significant positive relationships were between ACT and K10 change (*r =* .188) and Other interventions and K10 change (*r =* .116). A negative relationship was evident between Narrative therapy and LSS change (*r =* −.123). All relationships were weak.

**Table 4:** Pearson Correlations Between Number of Sessions and Change in Outcome Measures, by Intervention

Intervention	K10	SOFAS	LSS
CBT	.029	−.005	−.035
Behavioral	.028	.001	.002
Supportive Counselling	.001	−.038	.062
Crisis	−.006	.010	−.036
Psychoeducation	.053	.027	.045
IPT	.061	−.071	−.066
ACT	.188*	−.028	−.030
Mindfulness	.011	.037	−.009
Motivational Interviewing	.039	−.001	−.026
Narrative	−.021	−.075	−.123*
Other	.116*	−.010	−.065

*Notes. N* = 626, *p < .05

## Discussion

This study investigated the therapeutic interventions being used by clinicians working in early intervention mental health services, treating young people with a diagnosis of BPD and identified traits of BPD. Results revealed that as hypothesised, young people received a range of interventions with CBT being the most commonly used modality of intervention. CBT was the clear leader in treatment type, with supportive counselling and IPT being the next most commonly used. Given the lack of evidence related to traditional CBT, IPT or supportive counselling with this client population, these findings indicate concern regarding the implementation of effective intervention approaches. It was expected that clinicians would not be using evidence-based treatments with this client group, given the lack of treatment guidelines available at the time.

As anticipated, CBT was the most commonly used treatment approach, likely reflecting the training of clinicians and the evidence available at the time of data collection. The use of CBT with this population did not result in significant improvement in psychological distress, however. Results showed that the amount of CBT a client received did not correlate with significant positive change on any of the outcome measures. The results also revealed that only a very small number of clients (*n* = 2) received a full 10 sessions of CBT; almost no clients received a sufficient dose of CBT and most had a range of different types of interventions. Eclectic practice is common in real-world settings and well established for adult community-based mental health services (e.g., [26]). There is less evidence about variability in usual practice in youth mental health settings, but the current study suggests that clinicians need to adapt their practice to ensure engagement of young people and match their approach with the client’s current presenting needs. Qualitative research with adolescent and young adult clients, generally, revealed the need to vary practice to young people’s changing presenting issues to facilitate therapeutic change [17]. Similarly, clinical interviews with French adolescents with BPD and their families highlighted that a lack of adaptation of services to the needs of young people with BPD resulted in greater dropout, with almost half of suicidal adolescents with BPD dropping out of treatment [16].

Traditional CBT does not treat the core challenges of BPD presentations, including self-harming behaviours, contacts with emergency services, functioning or interpersonal effectiveness [14]. Early intervention modalities should specifically target key symptoms such as poor emotion regulation, identity, interpersonal effectiveness and self-injurious behaviour. DBT, which has good evidence in adult populations, and emerging evidence for adolescents, directly addresses these areas of dysfunction [27].

DBT with suicidal adolescents (DBT-A), when delivered with fidelity, requires the comprehensive implementation of treatment modes such as a skills group, individual therapy, phone coaching and family sessions [36]. However, given the imperative that early intervention needs to be brief, cost effective and accessible [11], and that young people accessing headspace services typically do so for fewer than six sessions, DBT-A, in its current comprehensive form, is unlikely to be able to be implemented with fidelity. Although DBT was not originally developed for early intervention, factors associated with the development of BPD, such as emotion dysregulation and chronic and pervasive invalidation [27], may be amenable to change even by using less comprehensive adapted DBT programs.

Engagement of young people in primary care mental health services is a challenge, with up to 20% dropout being evident at each subsequent session [39]. Importantly, however, this same research also showed that a quarter of headspace clients who had disengaged from an episode of care returned at a later timepoint. This means that manualised intervention approaches are generally not able to be implemented in practice as originally designed, and approaches need to be tailored to the typical pattern of young people engaging for brief, and possibly repeated, periods of time.

Recent research provides growing evidence that simpler, shorter ‘generalist treatments’, that do not require as much specialist training of clinicians, may be particularly appropriate and more realistic for such client groups [10]. Given that young people have unique challenges engaging in treatment and in accessing specialist treatments, greater use of generalist models may be warranted [12]. For example, the generalist model ‘Good Clinical Care’, which was designed specifically for young people, demonstrated very similar improved outcomes to the specialist psychotherapy (CAT) it was compared to [8].

It should be noted that the headspace youth mental health initiative was set up originally to provide early intervention for young people showing early symptoms and sub-syndromal presentations [37], and briefer, generalist approaches are more likely to be feasible and appropriate in a primary mental health care model. Nevertheless, it is evident that young people with more severe and persistent conditions, and with very high levels of psychological distress and poor quality of life, are presenting for care, and these young people are likely to need more intensive and longer-term care models [30].

Research also indicates that the earlier a young person presenting with BPD symptoms gets appropriate treatment, the more likely they are to have a reduction or become sub-clinical in their symptomatology in adulthood [21]. It is believed that the younger the client is, the more open they will be to engaging with treatment, a major indicator of treatment success. Early intervention is critical to outcomes for this client population and needs to be informed by research designed for these settings and the specific challenges of this diagnosis [11].

### Limitations

This study analyzed data routinely collected through a MDS from *headspace* youth mental health centers across Australia. As such, although it yielded a large data set on this client group, the information collected was not tailored to the aims of the study, which were fitted retrospectively. Importantly, the service types that clinicians could choose from were limited and use of the “other” option available meant that a proportion of the clinical interventions were unknown. DBT and Schema Therapy are notable omissions given the stronger evidence base for these treatments of BPD in adult populations. Further, as the results come from a real-world observational study, it is not known on what basis clinicians determined their diagnosis. This was ascertained according to usual practice, which was not defined and unlikely to be consistent, although categorization in the MDS was according to DSM-5 criteria. It should also be emphasized that few clients received a full course of any type of intervention and thus conclusions related to the efficacy of evidence-based interventions are not possible. Nevertheless, this study represents a snapshot of the interventions being received by this client population in real-world youth mental health settings, in the absence of current evidence-based treatment guidelines for this age range for this critical mental health condition.

### Directions for future research

The evidence base for treating BPD presentations in young people continues to grow. So, too does the headspace MDS, with more centers opening and over 100,000 young people accessing services each year [23]. A revised version of the MDS was implemented in late 2019 and improved measures for diagnosis and treatment type were included. As this large national network dataset develops, we will be able to more fully investigate the types of treatments provided and outcomes being achieved for young people at risk of BPD, and also will be able to drill down into differential impacts by age, gender and other potentially relevant client characteristics. Such analyses will help to understand how interventions for this client group are being implemented in the real world on a large scale in community mental health settings. Further, modalities that can be feasibly and meaningfully implemented in early intervention settings, for example those that are brief, cost effective and accessible, could result in better outcomes for young people meeting the criteria for BPD. The development of clear treatment guidelines addressing emerging symptoms of BPD has been highlighted as an area of future study [11]. Guidelines exist for other prominent diagnoses in early intervention, such as psychosis and mood disorders, but are yet undeveloped in BPD. Targeted research with this client population in early intervention settings is critically needed to establish these.

## Conclusion

The current study investigated the types of interventions clinicians provide young people meeting the criteria for BPD traits accessing early intervention youth mental health services in Australia. The study highlights that a wide range of interventions are being used, most of which have a limited evidence base. This may reflect that this challenging psychiatric condition lacks clear treatment guidelines for younger populations, particularly in the context of brief interventions suitable for primary mental health care settings. Given the major impact that this diagnosis has on young people’s lives and futures, and that it is mostly likely to emerge during adolescence, more effort is needed to strengthen the evidence base for early intervention treatments in primary mental health care and finding ways to support clinicians to provide interventions that are engaging and effective for young people.

## Data Availability

The data that support the findings of this study are available from *headspace* youth mental health centres, but restrictions apply to the availability of these data, which were used under license for the current study, and so are not publicly available.
